# Prevalence of MASLD in People With Diabetes Has Decreased in the US, but Rates of Liver Fibrosis Are Still on the Rise

**DOI:** 10.1210/jendso/bvaf110

**Published:** 2025-06-14

**Authors:** Fernando Bril, Parameshwara Bolla, Ky Huynh, Sumedha Bobba

**Affiliations:** Division of Endocrinology, Diabetes and Metabolism, Department of Medicine, University of Alabama at Birmingham, Birmingham, AL 35205, USA; UAB Comprehensive Diabetes Center, University of Alabama at Birmingham, Birmingham, AL 35205, USA; Division of Endocrinology, Diabetes and Metabolism, Department of Medicine, University of Alabama at Birmingham, Birmingham, AL 35205, USA; Division of Endocrinology, Diabetes and Metabolism, Department of Medicine, University of Alabama at Birmingham, Birmingham, AL 35205, USA; University of Alabama at Birmingham Heersink School of Medicine, Birmingham, AL 35205, USA

**Keywords:** NAFLD, NASH, cirrhosis, liver fat, obesity

## Abstract

**Objective:**

We aimed to evaluate recent changes in the prevalence of steatotic liver disease, its subtypes, and liver fibrosis among US adults with and without diabetes between 2017-2020 and 2021-2023.

**Research Design and Methods:**

In this serial cross-sectional study, we assessed adults aged ≥18 years with complete vibration-controlled transient elastography (Fibroscan®) data from the National Health and Nutrition Examination Surveys from 2017-2020 and 2021-2023. Metabolic dysfunction-associated steatotic liver disease (MASLD) was defined as liver steatosis in the presence of 1 cardiometabolic risk factor.

**Results:**

A total of 13 153 adults (n = 7683 from 2017-2020; n = 5470 from 2021-2023) were included. The prevalence of MASLD significantly decreased from 37.6% to 32.5% in this period (*P* = .001), and this was particularly noticeable among individuals with prediabetes (from 49.1% to 41.8%; *P* = .002) or diabetes (from 69.4% to 61.4%; *P* = .013). Despite the lower prevalence of MASLD, the prevalence of clinically significant fibrosis (≥F2), advanced fibrosis (≥F3), and cirrhosis (F4) significantly increased in this period, especially among individuals with diabetes. Of note, among individuals with diabetes, clinically significant fibrosis reached 27.4% and cirrhosis 10.2% in 2021-2023. Alcohol-related liver disease also increased from 1.0% to 1.7% (*P* = .037) in the overall population, with similar increases in prediabetes and diabetes.

**Conclusion:**

Despite the evidence of a decline in MASLD prevalence, rates of liver fibrosis and cirrhosis are rising, especially in individuals with diabetes. This underscores the need for improved screening of liver fibrosis, early initiation of effective pharmacological therapies, and aggressive management of related cardiometabolic conditions in individuals with diabetes.

Driven by the obesity epidemic and an increasing prevalence of type 2 diabetes, metabolic dysfunction-associated steatotic liver disease (MASLD) has become a serious public health problem [1]. Its presence is associated with increased fatal and nonfatal cardiovascular events (hazard ratio 1.45 [1.31-1.61]) [2], liver-related mortality (rate of 0.15 per 100 person-year) [3], and even extra-hepatic cancers (∼1.2-2.0 times higher, depending on cancer type) [4, 5]. This translates into a high economic burden due to direct and indirect healthcare-related costs [6]. Understanding the trends in prevalence is essential for guiding public health policies and prioritizing resource allocation.

A recent meta-analysis reported that the worldwide prevalence of MASLD among adult individuals went from 25% in 1990-2006 to 38% in 2016-2019 [7]. Similar increasing trends have been observed among adult individuals with diabetes, with a recent meta-analysis reporting a worldwide increase in the prevalence of MASLD from ∼56% in 1990-2004 to ∼69% in 2016-2021 [8]. Based on these and other data, several studies have attempted to model the predicted prevalence of MASLD in the near future [9, 10]. In general terms, these studies have agreed in predicting a continuous increase in the prevalence of MASLD and liver fibrosis in the foreseeable future. However, recently released data by the Centers for Disease Control and Prevention from the National Health and Nutrition Examination Survey (NHANES) showed that between 2021 and 2023, the prevalences of obesity and diabetes did not significantly change compared to 2017 to 2020 [11, 12].

In addition to MASLD (defined as the presence of hepatic steatosis in the presence of 1 cardiometabolic risk factor in the absence of other liver etiologies) and alcohol-related liver disease (ALD) [13], a new subtype of steatotic liver disease (SLD) was recently introduced to classify patients with MASLD who also consume moderate amounts of alcohol. This entity was called metabolic and alcohol related/associated liver disease (MetALD) [13], and its epidemiology and trends over time are largely unknown. Indeed, while prior work assessing recent trends in prevalence has focused on either SLD or MASLD, MetALD and ALD have been largely overlooked. It was crucial to appropriately include these SLD subtypes, especially in light of significant changes in alcohol consumption reported before vs after the COVID-19 epidemic [14]. Moreover, given the well-recognized impact that the presence of type 2 diabetes has on the natural history and progression of MASLD [15], it was also important to assess prevalence trends separating patients with vs without diabetes.

This study aimed to estimate the latest prevalence of SLD and its subtypes (ie, MASLD, MetALD, ALD), as well as liver fibrosis in an unselected cohort representative of the US population. Specifically, this study assessed the changes in prevalence of liver disease between 2017-2020 and 2021-2023 in individuals without prediabetes or diabetes, those with prediabetes, and those with diabetes.

## Methods

### Study Design and Individuals

This study is based on NHANES data collected from 2017-2020 and 2021-2023 [16]. NHANES is designed to select a representative sample of the general US population. It is a multiyear, stratified, clustered 4-stage sample of the US civilian noninstitutionalized population. Each cycle (ie, 2017-2020, 2021-2023) included 30 primary sampling units made up of counties from which area segments and then dwelling units were sampled. Participants were selected from within households in dwelling units as detailed elsewhere [17]. Due to the COVID-19 pandemic, data collection in 2020 was interrupted, leading to a 2019-2020 sample that was not representative of the United States. For that reason, NHANES released a unified 2017-2020 database. We compared results from this database against the recently released 2021-2023 database. The survey was approved by the Centers for Disease Control and Prevention Research Ethics Review Board, and written informed consent was obtained from all adult participants. All research procedures were conducted in accordance with the Declarations of Helsinki and Istanbul.

Individuals were included in the study if they were adult individuals (≥18 years old) and had complete data on vibration-controlled transient elastography (VCTE; which is used to estimate liver stiffness measurement, a surrogate of liver fibrosis) and controlled-attenuation parameter (CAP; which is used to estimate hepatic steatosis) by Fibroscan®. They were excluded if they reported other specific etiologies of liver disease other than metabolic or alcohol related (ie, autoimmune hepatitis or viral hepatitis). A diagram with the number of individuals excluded and the reasons for exclusion can be found in Supplementary Fig. S1 [18].

### Definitions

SLD was defined as CAP ≥ 274 dB/m as previously done by our and other groups [19, 20]. The presence of cardiometabolic risk factors and self-reported alcohol use was used to subclassify individuals into MASLD, MetALD, and ALD based on current criteria as defined here [13]. Among individuals with available alcohol self-reporting, MASLD, MetALD, and ALD were defined based on the presence of hepatic steatosis and according to their estimated alcohol use (ie, MASLD: weekly alcohol consumption <140 g/week [females] or <210 g/week [males], MetALD: 140-350 g/week [females] or 210-420 g/week [males], and ALD: > 350 g/week [females] or >420 g/week [males], respectively) [13]. Alcohol consumption was estimated assuming 14 grams per standard drink as previously done [21]. The diagnosis of MASLD and MetALD required at least 1 cardiometabolic risk factor as previously defined [13]. Sensitivity analyses using CAP ≥ 288 dB/m as the cut-off point are also presented [22].

Clinically significant fibrosis (F2-F4), advanced fibrosis (F3-F4), and cirrhosis (F4) were defined as a liver stiffness measurement (LSM) ≥ 8.2, ≥ 9.7, and ≥ 15 kPa, respectively [23, 24] based on VCTE or FIbroscan®. Sensitivity analyses using other cut-off points for LSM (eg, 8, 10, or 20 kPa) are also presented. The presence of diabetes was defined based on the presence of prior history, a hemoglobin A1c (A1c) ≥ 6.5%, or fasting glucose levels ≥ 126 mg/dL. Prediabetes was defined based on the presence of A1c between 5.7% and 6.4%. The presence of overweight and obesity were defined as body mass index (BMI) ≥ 25 kg/m^2^ and BMI ≥ 30 kg/m^2^, respectively, except among non-Hispanic Asians, where different BMI cut-off points were used (ie, BMI ≥ 23 kg/m^2^ and BMI ≥ 25 kg/m^2^, respectively).

### Analytical Measurements and Transient Elastography

Laboratory measurements included total cholesterol, high-density lipoprotein cholesterol, complete blood count with platelets, A1c, insulin, and fasting plasma glucose. They were performed as previously described [16]. VCTE and CAP were performed by NHANES health technicians as previously described following manufacturer's guidelines, with no differences in modality between the 2 time periods. Examiners were trained to take 10 valid measurements with an interquartile/median ratio <30% only on individuals fasting for 3 hours or more [25, 26].

### Statistical Analysis

Data are presented as percentages (categorical variables) or as mean ± SE (continuous variables). Weights provided in the NHANES database were combined to obtain estimates generalizable to the US population. When specified, estimates were also adjusted by age based on data from the 2000 US census using the following age groups: 18 to 24, 25 to 34, 35 to 44, 45 to -64, and 65 or over as previously reported [27]. Comparisons between NHANES databases were done by linear regression or chi-squared depending on variable type. Due to the COVID-19 pandemic, there was a gap in collection between the 2017-2020 and 2021-2023 databases. The assumption of our approach is that unobserved data between these 2 cycles (from April 2020 to June 2021) are not significantly different from observed data. As sensitivity analysis, we have provided multiple imputation using multinomial logistic regression to classify as MASLD, MetALD, ALD, or cryptogenic SLD those individuals with SLD but without alcohol self-report data, based on age, sex, and race. A 2-tailed value of *P* < .05 was considered to indicate statistical significance. Analyses were performed using Stata 18.5 (StataCorp LP, College Station, TX, USA), and graphs were generated with Prism 10.4.1 (GraphPad Software, Inc., La Jolla, CA, USA).

## Results

### Study Population

Table 1 summarizes individuals' characteristics in 2017-2020 and 2021-2023. As can be observed, clinical characteristics were well balanced in both databases with no significant differences in age, sex, race distribution, BMI, or A1c. Of note, prevalences of obesity and diabetes were not significantly different in 2017-2020 compared to 2021-2023 (42.4% vs 40.4%, *P* = .28; and 13.8% vs 13.8%, *P* = .98, respectively). Liver fat accumulation measured by CAP was also similar in both cohorts. Despite similarities in cardiometabolic risk factors and liver steatosis, LSM by transient elastography was significantly lower in 2017-2020 compared to 2021-2023 (5.7 ± 0.1 vs 6.1 ± 0.1 kPa, *P* = .026). Self-reported alcohol consumption significantly increased in this period from 51 ± 2 to 58 ± 2 g/week, *P* = .014, equivalent to a mean increase in half a drink per week. As shown in Supplementary Fig. S1 [18], a higher percentage of individuals had incomplete CAP and/or VCTE data in 2021-2023 compared to 2017-2020 (32.2% vs 18.9%, respectively, *P* < .001). There were no differences in clinical characteristics (age, sex, BMI, A1c) in individuals with incomplete VCTE or CAP data between 2017-2020 and 2021-2023. Of note, compared to individuals with complete data, those with incomplete VCTE/CAP data were younger and leaner.

**Table 1.** Individuals' demographic and clinical characteristics

	NHANES 2017-2020 (n = 7683)	NHANES 2021-2023 (n = 5470)	*P*-value
Age, years	47 ± 1	47 ± 1	.85
Sex, male/female, %	49/51	50/50	.87
Race, %			.44
Mexican-American	8.8	7.3	
Other Hispanic	7.7	9.5	
Non-Hispanic White	62.3	60.1	
Non-Hispanic Black	11.2	10.5	
Non-Hispanic Asian	5.9	6.3	
Other or multiracial	4.1	6.2	
Weight, kg	83.3 ± 0.5	83.0 ± 0.7	.68
Body mass index, kg/m^2^	29.4 ± 0.2	29.2 ± 0.2	.56
Waist circumference, cm	99.6 ± 0.5	99.2 ± 0.6	.57
Presence of overweight/obesity, %	72.9	71.2	.29
Presence of obesity, %	42.4	40.4	.28
Presence of prediabetes, %	19.4	19.0	.75
Presence of diabetes, %	13.8	13.8	.98
A1c, %	5.65 ± 0.02	5.67 ± 0.03	.52
Fasting plasma glucose, mg/dL	109 ± 1	108 ± 1	.45
Fasting plasma insulin, μU/mL	14 ± 0	13 ± 1	.66
Total cholesterol, mg/dL	187 ± 1	188 ± 1	.55
HDL-C, mg/dL	54 ± 0	54 ± 0	.98
SBP, mmHg	122 ± 0	121 ± 0	.095
DBP, mmHg	74 ± 0	75 ± 0	.118
CAP, dB/m	263 ± 1	261 ± 2	.41
Liver stiffness by VCTE, kPa	5.7 ± 0.1	6.1 ± 0.1	.026
Alcohol consumption (g/week)	51 ± 2	58 ± 2	.014

Abbreviations: A1c, hemoglobin A1c; CAP, controlled attenuation parameter; DBP, diastolic blood pressure; HDL-C, high-density lipoprotein cholesterol; NHANES, National Health and Nutrition Examination Survey; SBP, systolic blood pressure; VCTE: vibration-controlled transient elastography.

### Prevalence of SLD and Its Subtypes

**Figure 1.**
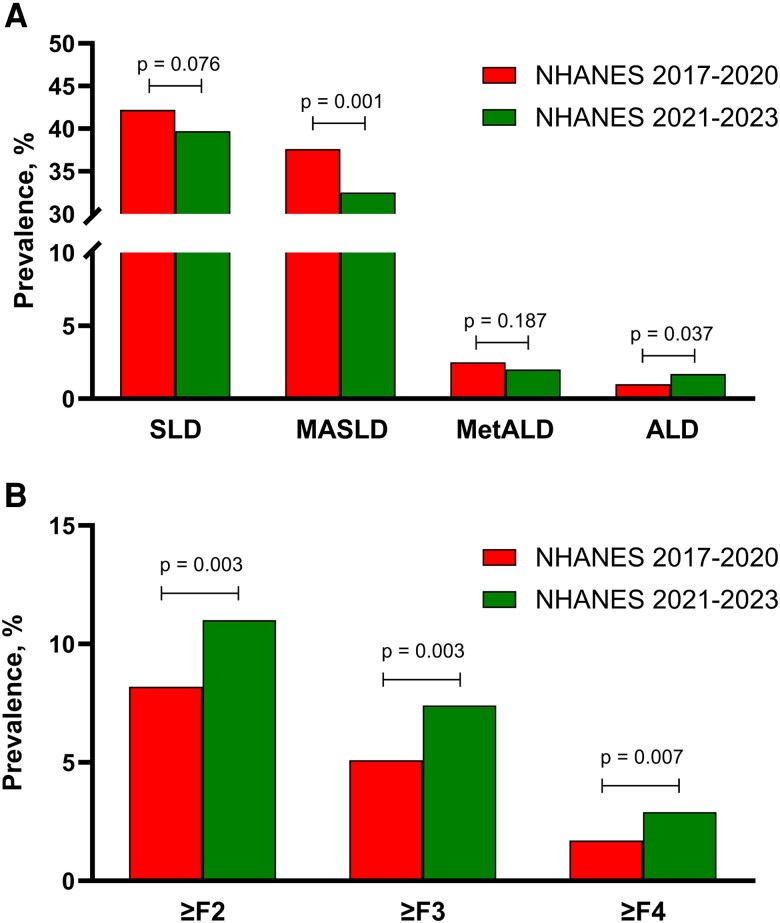
Weighted prevalence of SLD and its subtypes (MASLD, MetALD, and ALD) in 2017-2020 and 2021-2023 (A); and weighted prevalence of clinically significant fibrosis (≥F2), advanced fibrosis (≥F3), and cirrhosis (F4) in 2017-2020 and 2021-2023 (B). Abbreviations: ALD, alcohol-related liver disease; MASLD, metabolic dysfunction-associated steatotic liver disease; MetALD, metabolic dysfunction and alcohol-related liver disease; SLD, steatotic liver disease.

Overall, the prevalence of SLD individuals was not significantly changed from 2017-2020 to 2021-2023 (42.2% and 39.7%, respectively, *P* = .076; Fig. 1A). When looking specifically into MASLD, its prevalence significantly decreased from 37.6% to 32.5% (*P* = .001). No significant differences were observed in the prevalence of MetALD in 2017-2020 vs 2021-2023, while the prevalence of ALD almost doubled from 1.0% to 1.7% (*P* = .037). Of note, the prevalence of cryptogenic SLD was overall low but also significantly increased from 0.1% to 0.3% (*P* = .017). The number of participants who did not self-report alcohol consumption and were therefore excluded from the MASLD, MetALD, ALD, and cryptogenic SLD subclassification increased from 1.7% to 5.1% (*P* < .001). When applying multiple imputation for individuals with missing data on alcohol consumption, we did not see any significant differences compared to the analyses provided earlier for the prevalence of MASLD, MetALD, or ALD (data not shown).

### Prevalence of Liver Fibrosis

There was a significant increase in the prevalence of clinically significant fibrosis (≥F2), advanced fibrosis (≥F3), and cirrhosis (F4) in 2021-2023 vs 2017-2020 (Fig. 1B). Of note, the weighted prevalence of clinically significant fibrosis in the United States reached 11.0%, implying that ∼24 000 000 individuals had clinically significant fibrosis in the United States in 2021-2023. Among patients with clinically significant fibrosis, the etiologies were MASLD (73.5%), MetALD (5.6%), and ALD (6.6%); 13.7% could not be classified due to lack of alcohol self-reporting information. Our results also imply that ∼6 000 000 individuals had cirrhosis in the United States in 2021-2023. Of these, the etiology of cirrhosis was MASLD in 72.8%, MetALD in 3.6%, and ALD in 7.1%; 15.0% could not be classified due to lack of alcohol self-reporting information.

### Prevalence of SLD and Its Subtypes in Individuals With and Without Diabetes

**Figure 2.**
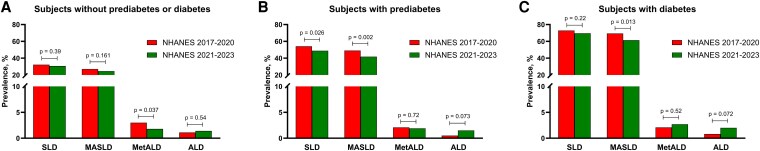
Weighted prevalence of SLD and its subtypes (MASLD, MetALD, and ALD) in 2017-2020 and 2021-2023 among individuals without prediabetes or diabetes (A), those with prediabetes (B), and those with diabetes (C). Abbreviations: ALD, alcohol-related liver disease; MASLD, metabolic dysfunction-associated steatotic liver disease; MetALD, metabolic dysfunction and alcohol-related liver disease; SLD, steatotic liver disease.

As can be observed in Fig. 2A, no significant changes in the prevalence of SLD, MASLD, or ALD were observed in individuals without prediabetes or diabetes, but they had a small reduction in the prevalence of MetALD (from 3.0% to 1.8%, *P* = .037). In individuals with prediabetes (Fig. 2B) and type 2 diabetes (Fig. 2C), there was a significant decrease in the prevalence of MASLD from 49.1% to 41.8% (*P* = .002) and from 69.4% to 61.4% (*P* = .013), respectively. No significant changes were observed in the prevalence of MetALD and ALD in individuals with prediabetes or diabetes. The number of individuals not reporting alcohol consumption significantly increased in all subgroups (from 1.5% to 3.3%, *P* = .005 [no prediabetes or diabetes], from 1.7% to 6.3%, *P* = .003 [prediabetes], and from 2.4% to 10.5%, *P* < .001 [diabetes]).

### Prevalence of Liver Fibrosis in Individuals With and Without Diabetes

**Figure 3.**
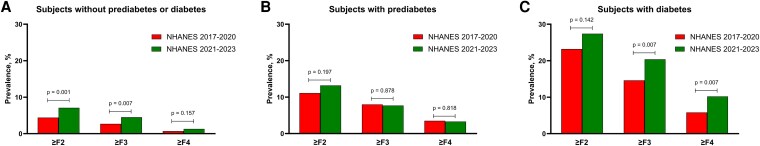
Weighted prevalence of clinically significant fibrosis (≥F2), advanced fibrosis (≥F3), and cirrhosis (F4) in 2017-2020 and 2021-2023 among individuals without prediabetes or diabetes (A), those with prediabetes (B), and those with diabetes (C).

In individuals without prediabetes or diabetes, we observed a small increase in the prevalence of clinically significant (≥F2) and advanced liver fibrosis (≥F3) when comparing 2017-2020 to 2021-2023 (Fig. 3A). Although the prevalence of cirrhosis in this subgroup almost doubled in absolute terms (from 0.7% to 1.3%), the overall prevalence was still low and not significantly affected. In individuals with prediabetes (Fig. 3B), we observed no significant changes in the prevalence of liver fibrosis (either clinically significant [ ≥ F2], advanced [ ≥ F3], or even cirrhosis [F4]). In individuals with diabetes, we observed a significant increase in the prevalence of advanced liver fibrosis and cirrhosis (Fig. 3C). The prevalence of advanced fibrosis among individuals with diabetes increased from 14.6% in 2017-2020 to 20.4% in 2021-2023 (*P* = .007). The prevalence of cirrhosis in diabetes increased ∼2-fold (from 5.8% in 2017-2020 to 10.2% in 2021-2023, *P* = .007). Overall, this represents ∼1 700 000 extra individuals with diabetes who were identified as having advanced fibrosis in this time frame, of which ∼1 300 000 were newly identified as having cirrhosis.

### Prevalence of Liver Disease After Age Adjustment and Other Sensitivity Analyses

Despite no difference in mean age between databases, we also adjusted weighted prevalences by age based on data from the 2000 US census as recommended [27]. The overall age-adjusted prevalence of SLD was 41.6% (2017-2020) and 39.2% (2021-2023) (*P* = .061). The age-adjusted prevalence of MASLD was 36.9% (2017-2020) and 32.1% (2021-2023) (*P* = .001), confirming a lower prevalence of MASLD in 2021-2023. The age-adjusted prevalence of clinically significant fibrosis (from 8.0% to 10.8%, *P* = .003), advanced fibrosis (from 5.0% to 7.1%, *P* = .004), and cirrhosis (from 1.7% to 2.7%, *P* = .006) increased from 2017-2020 to 2021-2023, respectively.

Once adjusted for age, no differences were observed in the prevalence of SLD in individuals with diabetes (from 73.3% to 73.1%, *P* = .97) between 2017-2020 and 2021-2023 or the prevalences of MASLD (from 69.3% to 64.3%, *P* = .35) and MetALD (from 2.8% to 2.6%, *P* = .93). The age-adjusted prevalence of ALD significantly increased from 0.6% to 3.7% (*P* < .001). Regarding liver fibrosis, the age-adjusted prevalence in individuals with diabetes was not significantly different for clinically significant or advanced fibrosis. However, cirrhosis prevalence significantly increased from 5.7% to 10.0% (*P* = .041), even after adjusting for age.

Using a different cut-off to define liver steatosis (i.e, CAP ≥ 288 dB/m) reduced the prevalence in absolute numbers, without affecting the changes observed between 2017-2020 and 2021-2023. For SLD, the prevalence was 34.1% (2017-2020) and 32.7% (2021-2023) (*P* = .30), and for MASLD, the prevalence decreased from 30.8% to 26.7% (*P* = .007). No significant changes were observed when a cut-off of 8 kPa LSM was used for clinically significant fibrosis instead of 8.2 kPa or if 10 kPa was used instead of 9.7 kPa for advanced fibrosis (data not shown). Using a cut-off of 20 kPa to diagnose cirrhosis, the overall prevalence of cirrhosis was not significantly changed in individuals with diabetes.

## Discussion

In the past few decades, we have witnessed a persistent increase in the prevalence of MASLD [7] with a higher incidence of liver-related complications [28]. However, results from this study suggest that in the United States, the prevalence of MASLD significantly decreased in 2021-2023 compared to 2017-2020. Specifically, this change appears to be driven by individuals with prediabetes and diabetes, a frequent target of current guidelines implementation efforts [29]. This marks an important finding, which provides the first evidence that we may have started to overturn the MASLD tsunami. However, this encouraging observation contrasts with the significant increase detected in the prevalence of clinically significant fibrosis, advanced fibrosis, and cirrhosis.

The highest increase in the prevalence of advanced liver fibrosis and cirrhosis was observed among patients with diabetes, emphasizing the particularly unfavorable natural history of MASLD in this subgroup of patients. As supported by our results, the progression of MASLD to liver fibrosis appears to be quite different depending on the glycemic status [30]. It is concerning that the prevalence of cirrhosis among individuals with diabetes almost doubled, with an estimated 10.2% of individuals with diabetes having cirrhosis in 2021-2023. While we decided to use 15 kPa as the cut-off to define cirrhosis [31], a more conservative approach with ≥20 kPa as the cut-off point still suggests that a shocking 6.1% of individuals with diabetes have cirrhosis. This contrasts with only 1.3% and 3.3% of individuals without prediabetes or diabetes or those with prediabetes, respectively. Surprisingly, we did not observe significant changes in the prevalence of liver fibrosis in individuals with prediabetes comparing 2017-2020 to 2021-2023, despite individuals without dysglycemia showing an increase in the prevalence of clinically significant and advanced fibrosis. The reasons for this are unclear, but it suggests that not all cardiometabolic risk factors may carry the same degree of liver fibrosis risk. Reports from our [32] and other [33] groups have shown that liver fibrosis risk is associated with hyperglycemia and A1c. Additional efforts targeting individuals with diabetes, as well as early detection and treatment of MASLD, may be warranted to start making a dent in halting the progression of liver fibrosis in the United States.

Other key findings of our study were the observed increase in ALD prevalence in the United States, as well as a reduction in MetALD among patients without prediabetes or diabetes. While a prior manuscript reported the prevalence of SLD subtypes in 2017-2020 with similar results to ours [34], no prior study looked at their prevalence change compared to data from 2021-2023. As the term MetALD has only recently been introduced, there is still much we do not know about its natural history and prevalence. Our study suggests that ∼2% of the adult US population had MetALD in 2021-2023. However, it should be recognized that this is likely an underestimation based on the fact that its definition is based on self-reported alcohol consumption. Of note, despite a significant decrease in MASLD prevalence, we observed a significant increase in ALD prevalence (from 1.0 to 1.7%, *P* = .037), mostly driven by changes in patients with prediabetes and diabetes. The COVID-19 pandemic may have played a role in some of these changes observed between 2017-2020 and 2021-2023 as increased alcohol consumption during and after the COVID-19 pandemic has already been reported [14]. Specifically, we observed a significant increase in self-reported alcohol consumption in this period from 51 ± 2 to 58 ± 2 g/week (*P* = .014), equivalent to a mean increase of half a drink per week. It is possible that some of the reduction in MASLD prevalence could be explained by differences in alcohol reporting and reclassification of individuals into MetALD or ALD.

The significant increase in the prevalence of individuals with clinically significant fibrosis, advanced fibrosis, and cirrhosis is worrisome. While recent guidelines have emphasized the importance of screening for liver fibrosis in individuals at risk (ie, those with diabetes, with 2 or more cardiometabolic risk factors, or with elevated alanine aminotransferase/aspartate aminotransferase or evidence of steatosis), implementation of these recommendations in clinics has been rather slow [35]. As a consequence of this, most individuals with liver disease are unaware of their ailment [36]. Our results suggest that ∼6 000 000 people may have cirrhosis in the US even if they are unaware of it. This contrasts with data from the National Health Interview Survey in 2018, where it was estimated that ∼4 500 000 people had chronic liver disease [37]. Raising awareness among individuals, but also among providers, should become a priority moving forward. Early identification and treatment of individuals with metabolic dysfunction-associated steatohepatitis and clinically significant fibrosis are of paramount importance to change the natural history of the disease.

The reduction in MASLD prevalence was observed despite no difference in the overall prevalence of overweight, obesity, or diabetes. Moreover, the difference between 2021-2023 and 2017-2020 persisted even after adjusting for age differences. Of note, the reduction in MASLD prevalence was only observed in individuals with prediabetes (∼7.3% reduction) or diabetes (∼8.0% reduction) compared to individuals without prediabetes or diabetes (no significant reduction). This may be the result of recent efforts to increase awareness of liver disease among individuals with prediabetes or diabetes, including MASLD guidance by the American Diabetes Association introduced in 2019 [38]. It is also possible that the larger improvement in individuals with prediabetes or diabetes is the result of the increasing use of glucagon-like peptide-1 receptor agonists (GLP-1RA) [39], which are more frequently prescribed in these individuals. However, no significant changes were observed in any of the subgroups regarding mean BMI or prevalence of overweight or obesity. While all GLP-1RA have shown improvement in hepatic steatosis, only tirzepatide (at 5, 10, and 15 mg weekly doses) and semaglutide 2.4 mg weekly have been shown to reduce liver fibrosis [40, 41]. Tirzepatide was only approved in 2022 for diabetes and at the end of 2023 for obesity. Semaglutide at higher doses was only approved in 2022 for diabetes (eg, at 2 mg weekly) and in 2021 for obesity (at the 2.4 mg weekly dose). Therefore, we can assume that the impact of this newer generation of GLP-1RA (ie, tirzepatide and higher doses of semaglutide) was only modest to induce changes between 2017-2020 and 2021-2023. Moreover, recent shortages and lack of coverage for obesity may have further limited the use of these drugs. In contrast, the use of other less potent GLP-1RAs in this timeframe may have contributed to improvement in steatosis without effects on liver fibrosis. Because the number of individuals not reporting alcohol consumption with SLD increased between 2017-2020 and 2021-2023 (from 1.7% to 5.1%), it is possible that by excluding these individuals, we could have affected the true prevalence of MASLD. However, performing multiple imputation to account for missing alcohol consumption data, we observed no differences in overall prevalence of MASLD, MetALD, or ALD.

While the significant reduction in MASLD prevalence is encouraging, this information should be interpreted carefully in light of the continuing increase in liver fibrosis. The apparent contradiction of higher rates of liver fibrosis despite the lower prevalence of MASLD likely responds to the relatively slow natural history of MASLD, the different rates of progression of steatosis and fibrosis, and their different response to therapies. Ectopic fat accumulation (steatosis) appears to be mostly driven by adipose tissue insulin resistance leading to increased free fatty acid supply [42] and increased hepatic de novo lipogenesis driven by hyperinsulinemia [43]. On the contrary, the drivers of liver fibrosis are less well understood, with genetic, epigenetic, and environmental factors playing significant roles [44]. While hepatic steatosis can fluctuate fast and responds to relatively minor lifestyle changes, changes in fibrosis require more time and more assertive approaches. For example, modest weight loss (≥5%) and a wide range of pharmacological therapies have been shown to significantly reduce steatosis, but only significant weight loss (≥10%) and few drugs have been shown to significantly improve liver fibrosis [45]. Given these facts, even if the latest efforts to increase MASLD awareness have succeeded in decreasing the prevalence of MASLD, it may be too soon to see the effects on liver fibrosis, which are likely to lag behind. Moreover, because time appears to be a key determinant for fibrosis progression [46] and prior reports have shown a persistent increase in MASLD prevalence in prior years [7], it is not surprising that the prevalence of liver fibrosis continues to increase. It is well established that increasing fibrosis stages is associated with a relative reduction in liver fat, and therefore our results could just reflect the natural history of worsening liver disease [47].

The strengths of this study are its large sample size and the fact that this population is representative of the overall US population. While a recent study used a similar database, it failed to explore the specific changes in individuals with or without diabetes or consider the prevalence of MetALD and ALD [48]. The main limitations of the study are the fact that the sample design and data collection for NHANES 2021-2023 was slightly different than NHANES 2017-2020 as there was no oversampling by race, Hispanic origin, or income, and therefore, sample sizes for subgroups may differ. Also, sample sizes were higher for people 60 years or older. Of note, even after adjusting for age, we observed similar results when comparing 2017-2020 to 2021-2023. No subgroup analyses were performed based on race, ethnic group, or income. Unfortunately, no information has been released on fasting triglycerides, serum alanine aminotransferase/aspartate aminotransferase, or the use of specific medications, and therefore, this information could not be included in the current manuscript. While noninvasive tests are widely accepted and recommended in guidelines for the diagnosis of liver disease, some misclassification can occur, especially in individuals with mild steatosis (for CAP) or only stage 2 fibrosis (for VCTE). We have provided data using different VCTE and CAP cut-off points, because these are not well established with some differences between guidelines [47, 49 , 50].

Alcohol consumption was based on self-report, and while this is subject to recall bias, it is the standard of care in clinical practice as other estimators of alcohol consumption, such as phosphatidylethanol levels, have not been standardized. Indeed, the current definitions of MASLD vs MetALD are based on self-reported amounts of alcohol intake [13].

In summary, recent data from NHANES shows that despite a significant decrease in the prevalence of MASLD, the proportion of individuals living with liver fibrosis continues to increase in the United States. Given their increased risk of developing hepatocellular carcinoma, requiring a liver transplant, liver-related mortality, and cardiovascular mortality [3], these findings should underscore the importance of implementing successful screening strategies, as well as early initiation of effective treatments aiming at reducing liver fibrosis and improving cardiometabolic risk factors. While the observed reduction in MASLD prevalence is encouraging, further efforts are needed to claim that we have started to turn the corner in the metabolic dysfunction-associated steatohepatitis epidemic.

## Data Availability

Original data generated and analyzed during this study are included in this published article [16].
